# A mixed‐methods evaluation of a health‐promoting café located in a small health service in rural Victoria, Australia

**DOI:** 10.1111/ajr.12901

**Published:** 2022-07-27

**Authors:** Jillian Whelan, Penelope Love, John Aitken, Lynne Millar, Catherine Morley, Ngareta Melgren, Steven Allender, Colin Bell

**Affiliations:** ^1^ Institute for Health Transformation, School of Medicine, Global Obesity Centre Deakin University Geelong Australia; ^2^ Institute for Physical Activity and Nutrition, School of Exercise and Nutrition Sciences Deakin University Geelong Australia; ^3^ LaTrobe Rural Health School La Trobe University Bendigo Australia; ^4^ Telethon Kids Institute Nedlands Western Australia Australia; ^5^ School of Population Health Curtin University Bentley Western Australia Australia; ^6^ Wimmera Health Care Group Horsham Victoria Australia; ^7^ Rural Northwest Health Service Warracknabeal Victoria Australia; ^8^ Institute of Health Transformation, School of Health and Social Development, Global Obesity Centre Deakin University Geelong Victoria Australia

**Keywords:** food environment, nudges, rural health, social enterprise, traffic light food classification system

## Abstract

**Introduction:**

Residents of rural areas internationally typically experience chronic disease risk profiles worse than city dwellers. Poor diet, a key driver of chronic disease, has been associated with unhealthy food environments, and rural areas often experience limited access to healthy, fresh and affordable food.

**Objective:**

This study aimed to evaluate the first three years of a health promoting social enterprise café established in a small rural health service.

**Design:**

A mixed‐methods evaluation study. Quantitative sales data, surveys and key informant interviews that included both quantitative and qualitative responses.

**Findings:**

Three years of sales data were included; 111customer surveys and five key informant interviews were conducted. Food and beverages on displayed and sold consistently met or exceeded the healthy criteria set by policy. Stakeholders supported the traffic light system, the social enterprise model and rated the likelihood of sustainability of the café as high.

**Discussion:**

Customers used the ‘traffic light’ system to inform food choices, placed value on the warmth of the staff and on the welcoming environment created through the social enterprise model. Resources remain tight although all stakeholders are committed to the sustainability of the YarriYak café.

**Conclusion:**

The study shows the acceptability, feasibility and sustainability of a health promoting social enterprise café in a rural area.

**What is already known on this subject:**

- Rural residents experience chronic disease risk profiles worse than residents of urban areas and poor diet is a leading cause of this chronic disease
- Poor diet has been linked with unhealthy food environments
- The food environment of this specific rural community was previously explored and found to provide limited access to healthy, fresh and affordable food, potentially exacerbating the existing health problems of the residents

**What this paper adds:**

- This study shows that residents of rural communities will purchase healthy food when it is available
- It adds to the evidence base that coding food according to the traffic light system can nudge people towards healthier choices
- A low‐resourced rural community can improve the healthiness of food environments through creative collaboration to provide options to their community

## 1 INTRODUCTION

Poor diet is a leading cause of the burden of disease globally and in Australia, resulting in multiple chronic diseases such as diabetes, heart disease and some cancers.^1^ Within Australia, most people do not meet national dietary guidelines,^2^ consuming too many energy‐dense, nutrient‐poor foods, sugar‐sweetened beverages and too few fruits and vegetables.^3^ This is particularly true in rural and remote areas of Australia, where residents often report poor dietary intake and experience chronic disease risk profiles worse than their metropolitan counterparts.^4^, ^5^ These rural and remote areas also experience limited access to healthy food.^6^, ^7^

Accessibility, availability and adequacy of food, collectively known as the food environment, in a community are influenced by physical, social, economic, cultural and political factors.^8^ Attempts to improve the healthiness of food environments include strategies that ‘nudge’ choices towards healthier options to address these factors, such as changes to the physical placement of products near checkouts, labelling and smaller portion sizes.^9^ These strategies have been shown to positively influence the buying intentions and dietary behaviours of customers.^10^ A recent review of the effect of food retail environments on diet by Mah et al.^11^ found 67% of 86 included studies described at least one positive effect on diet resulting from food retail environment interventions. Similarly, a review that investigated customer purchase intentions and choices in food retail environments concluded that shelf display (e.g., position of shelf), branding (e.g., familiarity) and nutrition labelling all influenced food purchases.^10^ Potential exists for health services to utilise these strategies to improve the healthiness of their internal food retail environments.

Health services in Australia and internationally have been criticised for providing or selling food that is energy‐dense and nutrient‐poor, thereby contributing to the chronic diseases they are funded to treat.^12^, ^13^ In an attempt to address this, governments in Australia have applied regulatory pressure to ensure these health services provide healthy food and drink choices to their workforces and their visitors.^14^ In Victoria, Australia, the State Government developed the Victorian Healthy Choices Classification Guide^14^; a suite of policy guidelines on product placement and labelling using traffic light colour coding based on nutrients such as saturated fat, sugar, sodium, fibre and kilojoules. Specific to health services, is the ‘Healthy Choices: Policy guidelines for hospitals and health services,^15^ hereafter referred to as ‘the policy’. This traffic light food classification system identifies ‘green’ options as the ‘best choice’, ‘amber’ options as those to ‘choose carefully’ and ‘red’ options as those that should be limited.^14^ The policy requires product displays to include at least 50% ‘green’ foods/beverages and no more than 20% ‘red’ foods/beverages, thereby promoting the consumption of a healthy diet.^15^

In rural areas, food‐related policies set by state Governments provide both opportunities and challenges. Specific to the rural context of the current study, an extensive food audit revealed a community food environment with an abundance of high‐fat takeaway foods, where access to healthy food was poor, and healthy choices, when available, were usually more expensive than their less healthy counterparts.^6^ The pivotal role rural health services play as community hubs^16^ and large local employers^17^ has the potential to facilitate leadership in the translation of these food‐related policies to practice in rural environments. This central role also facilitates partnership and engagement with other local organisations to further strengthen this leadership potential.

The state of Victoria, Australia is a leader in promoting partnership and engagement through social enterprises—a strategy that employs around 60 000 people and contributes $5.2 billion to the Victorian economy each year.^18^ Social enterprises are businesses that generate outcomes other than profit and aim to achieve multiple community benefits. They derive a substantial portion of their income from trade and are established to benefit the public or the community through economic, social, cultural or environmental missions. Importantly, the majority of their profits are used to fulfil their mission.^18^ Social enterprises not only provide economic and social benefits. There is also mounting evidence that they present opportunities for health promotion by themselves being a healthy workplace. They also seek to bridge the equity gap by impacting multiple levels of the socio‐ecological framework; individual, environment, workplace and policy.^19^ Given the socio‐economic gradient associated with obesity prevalence, any efforts to reduce these disparities can be positive steps towards reducing obesity.

This study describes the evaluation of a health‐promoting café established as a social enterprise within the grounds of a health service in an Australian rural community in response to a leadership‐led drive for healthier food provision, and desire to expand employment opportunities for clients of the local disability service (Woodbine). The café has the potential to benefit the whole population it serves, and the people and organisations that are directly involved. The day‐to‐day management is facilitated by Woodbine that provides hospitality staff and produces that is sold in the café.

The aim of this study was to evaluate the first three years of operations of the Café. The objectives were to:

1. establish the proportion of sales attributable to each of the categories of the traffic light system (red, amber, green);
2. determine customer perspectives of the ‘traffic light’ food classification system and the social enterprise model of the café;
3. ascertain sustainability of the café and of the use of the ‘traffic light’ food classification system.

## 2 METHODS

### 2.1 Setting

The study was conducted in the Yarriambiack Shire Council (YSC); a rural community located 360 km from the nearest State capital of Melbourne, Victoria, Australia. YSC has a population of 6658 people spread across 7326 square kilometres. Rural Northwest Health Service (RNH), located in Warracknabeal, the major town in YSC, employs 200 staff and, on an average day, has 35 allied health clients visit the premises. RNH also houses an onsite aged care facility; home to 60 residents.^20^ The Café (YarriYak Café) was located within the grounds of RNH as one component of a whole community systems approach to obesity prevention in a rural community.^21^ Within this broader prevention work, it was identified through a whole community food environment audit that it was difficult to access healthy food in this area.^6^ These data enhanced the motivation of the health service to establish this new health‐promoting café.

### 2.2 Evaluation

The evaluation comprised three types of data:

1. To measure compliance with the ‘Healthy Choices’ guidelines, product placement was assessed every 3 months for the first 18 months by in‐person audits of the food and beverages on display in the café (January 2016–July 2018) and a subsequent audit was conducted 8 months post the intervention period (March 2019). As a proxy for consumption, monthly sales data were obtained from the YarriYak Cafe cash register printouts. All sales data were captured from January 2016 to July 2018 (when researchers were active in the community, [intervention period]). Five months later, a further 3 months of sales data were collected (January 2019–March 2019) to assess the sustainability of the traffic light system and the social enterprise model of the cafe. The data comprised sales in dollars. Sales data were entered into an excel spreadsheet and analysed according to the colour‐coded food items: ‘green’ (e.g., vegetables, fruit, bread); ‘amber’ (e.g., dried fruit, fruit and vegetable juices with no added sugar, ham) and ‘red’ foods (e.g., sugars, traditional cakes, salami). These criteria are detailed in the Healthy Choices Food and Drink Classification Guide.^14^ Sales of each colour‐coded category were calculated as a percentage of total sales. Graphs were created using Tableau software.^22^
2. To determine staff and customer use of and satisfaction with the traffic light system and the social enterprise model, a survey was adapted from a tool previously applied in a scoping study for a ‘health hub’ in the United Kingdom^23^ (Appendix S1). The survey was administered to staff of the health service and customers of the café during July and August 2019 (hereafter called survey). The survey consisted of 35 questions; 19 of these were answered on an 11‐point Likert scale (0 = not at all interested, 10 = very interested), six were multiple‐choice, six were open‐ended and four were dichotomous responses (yes/no). The survey collected basic demographic data, participants' perceptions about various features of the café, the variety of food offered and the ‘traffic light’ system. The survey was administered in two ways: Firstly, an online version was sent by the internal staff email to all staff employed by RHS; secondly, to capture customers of the café who did not work in the health service, a researcher (JA) conducted researcher‐assisted online customer surveys at the café location. Survey data were analysed using Statistical Package for Social Scientists (SPSS Version 26). The data are presented using descriptive statistics. Independent sample *t*‐tests and chi‐square analyses were used to explore differences between groups. The level of statistical significance was set at *p* < 0.05.
3. To assess the sustainability of the traffic light system and the café, five key community leaders were purposively selected, based on their roles in the creation and continuation of the café, and invited, via email, to participate in interviews. All agreed to participate. One‐on‐one interviews were conducted in‐person, audio‐recorded, transcribed verbatim and thematically analysed. Interviews aimed to elicit key informant perspectives on the sustainability of using the traffic light classification system and the sustainability of the café itself (Appendix S2). The interview schedule contained 8 open‐ended questions and 10 questions using a 5‐point Likert scale (strongly disagree to strongly agree) regarding the 10 factors for sustainable obesity prevention (resourcing, leadership, workforce development, community engagement, partnerships, communication, policy, adaptation, evaluation and governance).^24^

The study received ethics approval from Deakin University HEAG‐H 80_2016.

## 3 RESULTS

### 3.1 Customer perspectives

#### 3.1.1 Traffic light system

Of the 123 customer surveys commenced, 111 (90%) were completed and used for analyses. Most participants were from the immediate area (*n* = 87; 78%) or the adjoining areas (*n* = 14%), most were female (72.6%; *p* < 0.001), around half were employed by RNH (56.1%), the largest age group were participants aged between 55 to 64 years (35%, *p* < 0.001).

More than one‐quarter (26%) of customers used the ‘traffic light’ system to inform their purchases all or most of the time. When combined with those who used the ‘traffic light’ system some of the time, the coding system influenced 56% of customers. This did not differ between females and males (Table 1). Full survey results are provided in Appendix S3.

**TABLE 1:** Use of the traffic light system to inform purchases in total and by sex‐specific mean values and standard deviations. There was no significance of differences between the sexes.

Use traffic light system to inform purchases	Total		Females		Males		*p*
	*N*	%	*N*	%	*N*	%	
All of the time	11	10.0	9	11.1	2	7.1	
Most of the time	18	16.2	15	18.5	3	10.7	
Some of the time	33	29.7	24	29.6	9	32.1	
No	49^a^	44.1	33	40.7	14	50.0	NSD
Total	111	100	81	100	28	100	

^a^ When stratified by sex, the numbers do not add to the total as two participants did not nominate sex.

Customers were asked what they most valued about the café with feedback, where offered, varying in relation to the traffic light system; for example, ‘The healthy options are great, [there is] no need for traffic light system, just provide fresh real food. Thank you’. Another response that stated the ‘traffic light system on food options’ was something they valued about the café, another expressed concern that ‘the older generation don't understand traffic light food’. Also, the range of options was noted: ‘The offering of healthy options but also having some red options available—it provides a balance’.

#### 3.1.2 Social enterprise

The support for the social enterprise partnership was strong with scores of over 8 from the range of 1–10 on the Likert Scales from the customer surveys (Table 2).

**TABLE 2:** Ratings of the partnership between the Woodbine and Rural Northwest Health Service with overall and sex‐specific mean values and standard deviations. There were no significant differences between sexes.

	Total (max 10)		Female		Male		*p*
	M	SD	M	SD	M	SD	
The partnership seems to work well	8.5	1.6	8.6	1.6	8.2	1.7	NSD
I am pleased that Woodbine provides the staff, and supplies the food and drinks for this café	8.8	1.7	8.9	1.8	8.6	1.5	NSD

Customer survey responses related to this social enterprise partnership were overwhelmingly positive: for example, ‘I think this has been a wonderful venture between RNH and Woodbine’, and ‘YarriYak goes well past a food outlet’. Others enjoyed ‘Good coffee and supporting a worthwhile initiative’. Several focused on the new opportunities created for people who experience a disability: ‘It shares resources and provides opportunities’. ‘Giving people a valued job where they may not be able to get one’ [Person with a disability] has come such a long way from the start.

#### 3.1.3 Unexpected outcomes

For staff of RNH, family and residents of the onsite aged care facility, the convenience of the location, feeling looked after and having a comfortable place to sit were particularly important features. Customers reflected on this: ‘Aged care residents and families can meet there, people who you wouldn't see unless you visited their ward’, and ‘[A] place to meet people, [I]can take my parents and they can spend time with aged care friends who they no longer see regularly’.

### 3.2 Sales data

**FIGURE 1:**
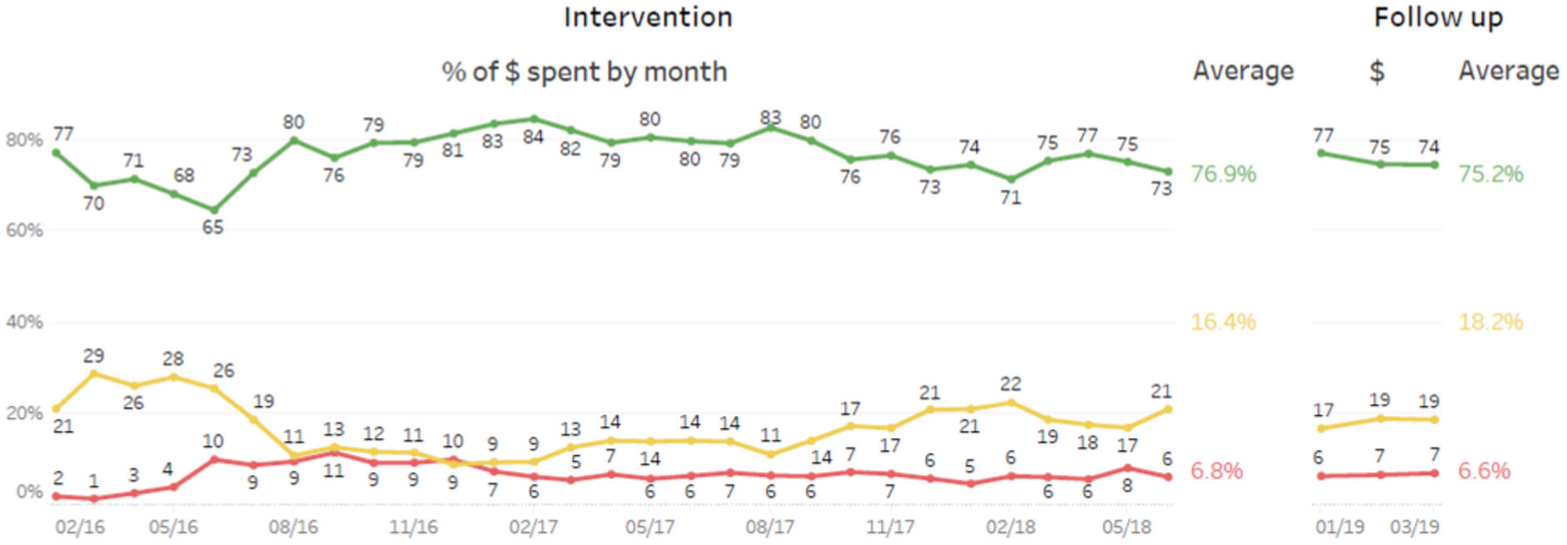
Sales data colour‐coded into categories as a percentage of total dollars of sales per month, *x*‐axis is date, *y*‐axis is percentage of sales.

Product placement audits found that the café was compliant at all time points with the prescribed ratios. The food retailer consistently displayed food and beverages as: 68% green, 12% amber and 20% red. In regard to the sales data, used as a proxy for consumption, Figure 1 shows the colour coding of sales per month with the lines green, amber and red representing the related sales of each category. Average ‘green’ sales in dollars during the intervention period (01/16–06/18) were 77% of total sales; ‘amber’ sales during the same period were 16% of total sales, ‘red’ sales were 7% of total sales. Three months later, (01/19–03/19) sales in dollars data showed ‘green’ sales made up an average of 75%, ‘amber’ sales 18% and ‘red’ sales 7% of total sales.

### 3.3 Sustainability of the café

Across the elements important for sustainability from the key informant perspective, the average score was 4.2 out of a possible 5 (Likert Scales) representing strong confidence that the café would be sustainable. Confidence was weakest for adaptation (the degree of flexibility allowed to change to the local context) with a score of 3.7/5.0 and strongest for leadership (mobilisation of capacity, interactions, innovation, collaboration and trust), which scored 4.5/5.0. (Appendix S4).

Key informants considered adaptation was hampered by the ability to source ingredients consistent with the healthy choices guidelines in this rural area, for example, low‐fat cheese and low sodium ham were not available from their regular supplier. Leadership scored highly due to the demonstrated commitment from both CEOs of the health service and the disability service. This leadership and commitment were acknowledged by all who participated in the key informant interviews. Available time and resources were acknowledged to be factors that required attention to ensure the sustainment of both the café and of the traffic light‐coding system.

## 4 DISCUSSION

This study explored sales and customer perceptions of a café within a rural health service that utilised traffic light labelling to nudge customers towards healthier choices. This study also examined the sustainability of the YarriYak Café. We found the display of healthy ‘green’ food and beverages consistently exceeded the policy requirements. Sales data were encouraging with an average of 77% of total sales of ‘green’ foods and beverages whilst red food and beverages comprised only 7% of sales throughout the intervention and follow‐up period. Secondly, we found the social enterprise model was widely endorsed by the customer base and key informants. Thirdly, after considering 10 published elements of sustainability, stakeholders were confident that the café would continue operating long‐term.

Product displays consistently met 68% green (healthy) criteria, compliant with the Victorian Healthy Choices guidelines.^25^ A recent audit at two major Sydney public hospitals found that ‘everyday’ foods (similar to ‘green’) comprised between 44% and 51% of foods displayed. Although our study compares favourably with these results, it may not meet the NSW Government guidelines^26^ requiring the display to comprise 75% of ‘everyday’ foods. Given that display audits did not directly align with sales in our study, a review of guidelines to comprise both product placement and sales audits may provide additional valuable insights as to purchasing behaviours. In one example, a large health service implemented the ‘traffic light’ system in 37 food and beverage vending machines located across three sites. Pre‐ and postimplementation of the ‘traffic light’ system, assessed by monthly sales, showed a reduction of 55% in sales of ‘red’ foods and 56% of ‘red’ drinks.^27^ In a different setting, audits of food and beverage vending machines in seven Australian Universities revealed improvements in the ratio of healthy to unhealthy foods between 2014 and 2017 in response to the introduction of the Health Star Rating on food packaging.^28^ Our data may similarly suggest that customers choose fewer red options when appropriate healthier options are provided. These studies align with the proposition that appropriate policy (regardless of specific measures, traffic lights, health stars, etc) can lead to changes to food environments and to food purchase behaviours, regardless of context.

Finally, in a sports‐centre setting, Boelsen‐Robinson et al.^29^ found that more than half of the food purchased from the café was ‘red’, increasing to 92% for children in a survey of 2326 people. These findings point to what sales may have been in the YarriYak café if the ‘20%’ maximum of ‘red’ foods/beverages had not been implemented from the start.

Internationally, the use of ‘traffic light’ coding for food choices has not been well reported. One US study of college cafeterias,^30^ utilising a pre–post intervention evaluation of ‘traffic light’ labelled foods, reported that 60% of customers found the labelling helpful, with 57% using them a few times a week. These results are comparable to our study with 56% of customers reporting them as helpful. A review of frameworks for restaurant owners to promote healthy food environments concluded nudge approaches appear more effective when supported by policy,^9^ as was the case in our study. Collectively, these findings add to the literature suggesting that overt labelling of healthy products helps nudge citizens towards these healthier choices,^31^ and that consumption is shaped by the food environment.^31^, ^32^

These findings align with concepts of ‘therapeutic landscapes’, proposed by Gesler (1992),^34^ where well‐being is promoted through the provision of spaces that impact people's health in positive ways. In the survey, YarriYak Café received high ratings for convenience and level of service, and several community respondents said they felt ‘special’ at YarriYak Café. Convenience was described in several ways such as it being a place to wait between appointments or while others had procedures, or to have a snack after a fasting blood test, and to get fresh healthy food (which is elsewhere reported to be scarce in this rural community^6^). These results, and the fact that the café is social enterprise, are consistent with YarriYak Café providing a place for socialisation or ‘therapeutic landscape’ before and after medical and allied health consultations. These findings are also consistent with how one defines success for social enterprises, namely, that they are accessible and relevant to their own communities as was demonstrated by YarriYak Café.^35^

The contribution of health service cafés to socialisation has been noted in other studies, for example ‘…[they] gave a feeling of normality and somewhere to go with families and visitors’ (p. 268).^36^ For inpatients, it is a de‐institutionalised space that allows people to interact without the rules and requirements that may occur on the ward. For aged care residents, it provides an opportunity for inter‐generational engagement allowing the resident ‘respite’ in the café environment,^37^ and young family members to feel more comfortable and less constrained than they would be in a room on an aged care ward. This may be contingent on the purpose of the café being consistent with the purpose of the setting; however, the presence of an international fast‐food chain in Melbourne^38^ and Auckland^39^ paediatric hospitals has caused controversy with many patients, staff and members of the public.

Despite the level of confidence about the café's sustainability, various challenges were identified throughout both the surveys and key informant interviews. These are frequently aligned with time and resource constraints, consistent with the deficit model of rural health elsewhere reported, where resources are invariably inadequate to meet the demand for a wide variety of localised services.^40^

In conclusion, the use of traffic light labels to nudge healthier choices to meet the ‘Healthy Choices: Policy for hospitals and health services’ can be utilised successfully within a health‐promoting café. Utilising the same strategy, it is also possible to provide food that is acceptable to staff, visitors and patients. Aspirational nutrition targets were continuously exceeded in regard to food and beverage sales. Customers used the ‘traffic light’ system to inform food choices, were mostly happy with the café and placed value on the warmth of the staff and on the welcoming environment created. Resources remain tight although all stakeholders are committed to the sustainability of the YarriYak café. The policy set by the Victorian Government provided the stimulus and framework for this to occur, but leadership, engagement and most importantly adequate resourcing are needed. Other rural health services would benefit from exploring the model of YarriYak Café and adopting a similar partnership model of a health‐promoting café within their sites.

## AUTHOR CONTRIBUTIONS

JW: conceptualization; investigation; methodology; resources; software; validation; visualization. PL: conceptualization; methodology; supervision; validation. JA: investigation; methodology. LM: conceptualization; methodology; supervision; validation. CM: conceptualization; investigation; methodology; resources; supervision. NM: conceptualization; investigation; resources; supervision. SA: methodology; supervision. CB: conceptualization; investigation; methodology; resources; supervision; validation; visualization.

## FUNDING INFORMATION

This research was funded by Rural Northwest Health, Warracknabeal, Vic, Australia; Royal Flying Doctors Service, Vic, Australia. JW is supported by the National Health and Medical Research Council (NHMRC)‐funded Centre of Research Excellence in Food Retail Environments for Health (RE‐FRESH) (APP1152968). The opinions, analysis and conclusions in this paper are those of the authors and should not be attributed to the NHMRC. JW is also supported by a Deakin University Dean's postdoctoral research fellowship. At the time this work was conducted, Allender was a Chief Investigator and researcher within the NHMRC Centre for Research Excellence in Obesity Policy and Food Systems (APP1041020). The opinions, analysis and conclusions in this paper are those of the authors and should not be attributed to the NHMRC.

## ETHICAL APPROVAL

This study received ethics approval from Deakin University HEAG‐H 80_2016.

## CONFLICT OF INTEREST

The authors declare no conflict of interest. The funders had no role in the design of the study; in the collection, analyses or interpretation of data; in the writing of the manuscript; or in the decision to publish the results.

## Supporting information


Appendix S1

Appendix S2

Appendix S3

Appendix S4
