# Supplementary material for: A mixed‐methods evaluation of a health‐promoting café located in a small health service in rural Victoria, Australia
Source: Aust J Rural Health. 2022 Jul 27;31(1):61–9. doi: 10.1111/ajr.12901 (PMC10946910; doi:10.1111/ajr.12901)
Supplement: Supplementary file 1 — Appendix S1 [file AJR-31-61-s004.docx]

Appendix I

YarriYak Customer survey

1. Firstly, we'd like to collect some general demographic data to help us analyse the data.
   1. What is your sex?
      1. Male
      2. Female
      3. Prefer not to answer
2. What is your age?
3. Do you work in Rural Northwest Health?
4. What is the postcode of the town where you live?
5. Have you ever bought food or drinks from YarriYak Café?
6. On a scale of 0 (not at all interested) to 10 (very interested), please indicate your level of interest in:
   1. Healthy eating
   2. Physical activity
7. Why do you have this interest in healthy eating and/or physical activity? (You may choose more than one answer.)
   1. To improve or maintain general health
   2. To feel better
   3. to get fitter
   4. I care about the health of my family
   5. My GP or health professional has advised me to do this
   6. to help me lose weight
   7. I am interested but I usually do not make healthy choices
   8. I am not interested in healthy eating
   9. I am not interested in physical activity
   10. other? (11) ________________________________________________
8. Do you want YarriYak café to continue operating?
   1. Definitely yes
   2. Yes
   3. Maybe
   4. No
   5. Don’t care
   6. Other __________________________
9. How important to you are each of these features of YarriYak café (0=not at all important, 10=very important)
   1. Healthy meals
   2. Healthy snacks
   3. Affordable prices
   4. The traffic light system to help choose healthy options
   5. The convenience of YarriYak in RNH
   6. Familiar range of foods
   7. A variety of foods
   8. Feeling looked after and welcome
   9. Good coffee
   10. Good range and quality tea
   11. It has become a routine/habit
   12. Having treat food available
   13. Having a comfortable place to sit
   14. It is a reward to a treat to eat and/or drink in the café.
10. Why do you come to the YarriYak café? (You may choose more than one answer.)
    1. I work at Rural Northwest Health
    2. I work close by
    3. I have just been to a doctors appointment
    4. I have just been to an allied health appointment
    5. I like to coffee here
    6. I like the café
    7. I like to support the partnership between Woodbine Disability Service and Rural Northwest Health
    8. It’s convenient
    9. Please add further comments ______________________________________
11. Do you use the traffic light system to inform your purchases?
    1. Yes, all of the time
    2. Most of the time
    3. Some of the time
    4. No
12. These questions relate to the partnership between Woodbine Disability Service and Rural Northwest Health. Please indicate your level of agreement with each statement. (1=completely disagree to 10=completely agree)
    1. The partnership seems to work well
    2. I am pleased that Woodbine provides the staff and supplies the food and drinks for this café.
    3. I am glad that YarriYak has created more employment opportunities for Woodbine.
13. What do you value most about the YarriYak café?
14. If you have any suggestions for improvements to YarriYak, please list these here.
15. If you have any further comments in regards to anything about the café, please add them here.
