# Supplementary material for: A mixed‐methods evaluation of a health‐promoting café located in a small health service in rural Victoria, Australia
Source: Aust J Rural Health. 2022 Jul 27;31(1):61–9. doi: 10.1111/ajr.12901 (PMC10946910; doi:10.1111/ajr.12901)
Supplement: Supplementary file 2 — Appendix S2 [file AJR-31-61-s001.docx]

Appendix II

Key informant interview questions

1. How do you communicate the healthy choices traffic light system to staff and customers?
2. What do you see as the big difference between YarriYak café and other cafes?
3. What kind of feedback have you received from customers?
4. What are the top selling menu items?
5. What new ideas/new menu ideas have been created since YarriYak café started?
6. What advice would you give to other cafes considering implementing the healthy choices guidelines?
7. Rate on a scale of 1 to 5 (1=strongly disagree to 5=strongly agree; or state not applicable)
   1. There adequate human resources to sustain the YarriYak café.
   2. There are adequate final resources to sustain the YarriYak café.
   3. Relevant Leaders within Rural Northwest Health are highly engaged to support the ongoing sustainability of the YarriYak café.
   4. Relevant leaders within Woodbine Disability Services are highly engaged to support the ongoing sustainability of the YarriYak café.
   5. Staff in YarriYak café have received adequate training in the traffic light system.
   6. Staff in Woodbine Disability Services kitchen have received adequate training in the traffic light system.
   7. Staff in Rural Northwest Health service have received adequate training in the traffic light system.
   8. Staff in YarriYak café have received adequate training in food safety.
   9. Staff in YarriYak café have received adequate training in coffee making.
   10. The broad community are highly engaged and supportive of the YarriYak café.
   11. The partnership between Rural Northwest Health and Woodbine Disability Services is well articulated in writing.
       1. Are there other key partners for YarriYak café?
   12. There are adequate communications between Rural Northwest Health and Woodbine Disability Services are clear.
   13. There is a formal communication plan for YarriYak café to promote our business.
       1. Examples of how communications could be improved.
   14. The policy related to traffic light coding is very clear.
   15. YarriYak Café has the ability to adapt to changes in customer demand.
   16. The governance structure of YarriYak café is very clear.
8. What do you consider the key elements that should be evaluated within the YarriYak café?
9. Are there any other recommendations you would like to make regarding the YarriYak café?
