# Supplementary material for: A mixed‐methods evaluation of a health‐promoting café located in a small health service in rural Victoria, Australia
Source: Aust J Rural Health. 2022 Jul 27;31(1):61–9. doi: 10.1111/ajr.12901 (PMC10946910; doi:10.1111/ajr.12901)
Supplement: Supplementary file 3 — Appendix S3 [file AJR-31-61-s003.docx]

Appendix III: Ratings of the levels of interest in participating in healthy activities, of the importance of café features, and of the partnership between the local disability and health services in total and by sex

|  | Total | | Females | | Males | |
| --- | --- | --- | --- | --- | --- | --- |
|  | M | SD | M | SD | M | SD |
| Level of interest in participating in:  (0 – not at all interested to 10 – very interested) |  |  |  |  |  |  |
| Healthy eating | 8.0 | 1.8 | 8.1 | 1.9 | 7.9 | 1.4 |
| Physical activity | 7.7 | 1.8 | 7.7 | 1.8 | 7.6 | 1.7 |
| Importance of café features: (0 – not at all important to 10 – very important) |  |  |  |  |  |  |
| Healthy meals | 7.7 | 2.0 | 7.7 | 2.1 | 7.6 | 1.9 |
| Healthy snacks | 7.5 | 2.2 | 7.7 | 2.2 | 7.0 | 2.5 |
| Affordable prices | 8.1 | 1.9 | 8.4 | 1.6 | 7.5 | 2.4 |
| Familiar range of food | 6.8 | 2.1 | 6.9 | 2.0 | 6.6 | 2.3 |
| Variety of food | 8.3 | 1.6 | 8.4 | 1.5 | 7.8 | 1.8 |
| Feeling looked after | 8.7 | 1.6 | 8.9 | 1.45 | 8.3 | 2.1 |
| Good coffee | 8.7 | 2.3 | 8.5 | 2.6 | 9.2 | 1.0 |
| Good tea | 6.0 | 2.9 | 6.4 | 2.9 | 4.7 | 3.1 |
| Traffic light system | 6.3 | 2.8 | 6.6 | 2.8 | 5.5 | 2.6 |
| Availably of treat foods | 6.0 | 2.9 | 6.4 | 2.8 | 4.9 | 2.8 |
| Comfortable place to sit | 7.7 | 2.3 | 8.0 | 2.2 | 6.8 | 2.2 |
| Convenience of location | 8.8 | 1.5 | 8.8 | 1.5 | 8.8 | 1.4 |
| Routine or habit | 6.2 | 2.8 | 5.9 | 2.7 | 6.9 | 2.9 |
| Reward to eat or drink there | 6.7 | 2.7 | 6.7 | 2.8 | 6.9 | 2.6 |
| Partnership between disability & health services: (1 - completely disagree to 10 - completely agree.) |  |  |  |  |  |  |
| I am pleased that disability service provides the staff and supplies the food and drinks for this café | 8.8 | 1.7 | 8.9 | 1.8 | 8.6 | 1.5 |
| I am glad that the cafe has created more employment opportunities for the disability service. | 9.2 | 1.4 | 9.3 | 1.4 | 8.8 | 1.4 |
