# Supplementary material for: A mixed‐methods evaluation of a health‐promoting café located in a small health service in rural Victoria, Australia
Source: Aust J Rural Health. 2022 Jul 27;31(1):61–9. doi: 10.1111/ajr.12901 (PMC10946910; doi:10.1111/ajr.12901)
Supplement: Supplementary file 4 — Appendix S4 [file AJR-31-61-s002.docx]

Appendix IV: Sustainability elements with relevant supporting statements, mean score (range)

| Element | Supporting statements | Mean (range) |
| --- | --- | --- |
| Resourcing | Support from both RNH and WDS Boards. Free rent and dietician support provided by RNH, catering and daily management provided by WDS.  Size of the ‘space’ of café may be considered a constraining resource.  Not ‘profitable’, but arguable that social enterprise benefits outweigh this and different ‘financial viability’ metrics could or should be applied. | 4.20  (range: 3.5-4.5) |
| Leadership | Initially, research driven by researchers, RNH and WDS CEOs and executives (champions). Dietitian has crucial leadership role (both champion and gatekeeper). | 4.50  (range 4.0-5.0) |
| Workforce development | WDS was trained in the traffic light system (by researchers). Jobs created for two (minimum) WDS clients. Improves catering skills and barista of WDS. Added health range to broader catering menu of WDS. | 4.30  (range: 3.5-5.0) |
| Community engagement | The predominant location of the café (next to the entrance) promotes community engagement. Promotion of Cafe in WDS monthly newspaper column. Promoting healthy eating at all RNH events. No sweet treats provided by external consultants. | 4.20  (range: 3.5-5.0) |
| Partnerships | The principal operating partnership is between RNH and WDS. The curry supplier is an integral partner in providing food one day per week. The researchers assisted with the initial set up and evaluation, but have no ongoing role. The ongoing partnership is managed at staff level between the catering manager of WDS, RNH Community Health Executive manager, the café manager and the RNH dietician. | 4.40  (range: 3.0-5.0) |
| Communication | The café is indirectly promoted to community through newspaper columns, presentation(s) at AGMs. The café utilises point of sale promotion of Traffic Light System provided. ‘Word of mouth is just the best thing.’ Minutes of meetings, emails, ‘putting things in writing helps’. Social Media (Facebook). Potential for real or perceived stigma against dietitian as the incumbent in the role felt pressure that ‘*nobody likes to be told what to eat’.* | 4.0  (range: 3.5-4.5) |
| Policy | Meets requirements of Victorian Government Policy Document: *Healthy choices: policy guidelines for hospitals and health services.*  Meets internal catering guidelines of RNH.  Meets WDS NDIS policy guidelines for job creation. | 4.40  (range: 4.0-5.0) |
| Adaptation | Curries have been included in the menu adapted to meet ‘traffic light criteria’ and are very popular. Broader implications include WDS catering has broadened menu to include healthy choices. Wider adaptation is limited by the availability of a dietitian and the real or perceived limitation of the policy guidelines. There is some demand to extend the menu to meet dietary requirements, eg coeliac. There is a time lag between feedback and implementation, for example specific ‘ham’ is required due to sodium content which is difficult to source in rural areas. A kitchen in the café would help with flexibility. Menu is planned a month ahead (WDS). | 3.70  (range: 3.0-4.0) |
| Evaluation | NDIS guidelines achieved. RNH and WDS staff need more training on “traffic light system” (staff turnover and complex guidelines). Concern regarding who will take over what [researcher] has done with colour coding of sales? Customers would like more variety and flexibility in food choices – constrained by resources and policy. Not enough time, and resources, for formal evaluation within RNH and WDS (rural deficit model).  Researcher evaluation comprehensive. | 4.0  (range: 0.0) |
| Governance | Handshake agreement at CEO level. Board chairs have championed the project. Regular bi-monthly committee meetings. Some reported the lines of reporting are not clear to all. Recent changes to executive roles at RNH have created uncertainty however new management has re-iterated support for YarriYak as a health promoting café. | 4.20  (range 4.0-5.0) |
| OVERALL SCORE |  | 4.19 (range 3.7-5.0) |
